# PARKA AI: A Sensor-Integrated Mobile Application for Parkinson’s Disease Monitoring and Self-Management

**DOI:** 10.3390/bioengineering12101059

**Published:** 2025-09-30

**Authors:** Krisha Sanjay Bhalala, Hamid Mansoor

**Affiliations:** Department of Computer Science, University of Manitoba, Winnipeg, MB R3T 2N2, Canada; bhalalk1@myumanitoba.ca

**Keywords:** Parkinson’s disease, mobile health, sensor integration, human-centered design, LLM, HealthKit, artificial intelligence

## Abstract

Parkinson’s disease (PD), a progressive neurodegenerative disorder affecting over 10 million people worldwide, necessitates continuous symptom monitoring to optimize treatment and enhance quality of life. Effective communication between patients and healthcare providers (HCPs) is vital but often hindered by fragmented data and cognitive impairments. PARKA AI, a novel iOS application, leverages Apple Watch HealthKit data (e.g., tremor detection, mobility metrics, heart rate, and sleep patterns) and integrates it with self-reported logs (e.g., mood, medication adherence) to empower PD self-management and improve patient–HCP interactions. Employing a human-centered design approach, we developed a high-fidelity prototype using a large language model (LLM)— Google Gemini 1.5 Flash—to process and analyze self-reports and objective sensor-derived data from Apple Healthkit to generate patient-friendly summaries and concise HCP reports. PARKA AI provides accessible data visualizations, personalized self-management tools, and streamlined HCP reports to foster engagement and communication. This paper outlines the derived design requirements, prototype features, and illustrative use cases to show how LLMs can be used in digital health tools. Future work will focus on real-world usability testing to validate the application’s efficacy and accessibility.

## 1. Introduction

Parkinson’s disease (PD) represents one of the most pressing challenges in modern neurological care, affecting approximately 1% of individuals over 60 worldwide, with prevalence projected to reach 12.9 million cases by 2040 [1,2]. This complex brain disorder exhibits considerable variation in how symptoms appear and progress, characterized by changing motor and nonmotor symptoms that differ substantially between patients and fluctuate within the same individual over time [3,4]. The unpredictable nature of PD symptoms, combined with its progressive course, demands innovative approaches to continuous monitoring and personalized treatment that transcend traditional care models based on periodic clinic visits.

The current healthcare system reveals fundamental communication problems between patients with chronic neurological conditions and their care providers, with particularly serious implications for PD management [5,6]. Patients experience significant difficulty in accurately remembering and describing symptom patterns during brief clinical appointments, while healthcare providers face increasing pressure to make complex treatment decisions based on limited, historical data [7]. These communication barriers are made worse by disconnected health information systems that fail to capture the time-based changes essential for understanding individual disease patterns and treatment responses.

Current digital health tools in the neurological field demonstrate promising but limited capabilities in addressing the comprehensive needs of PD care systems. While specialized applications have been developed for diagnostic purposes [8] and caregiver support systems [9], substantial gaps remain in developing integrated platforms that simultaneously support patient self-management and facilitate smooth clinical communication. The absence of comprehensive solutions that use both objective sensor-derived measurements and patient-reported outcomes represents a critical barrier to advancing personalized medicine approaches in PD care.

Recent technological advances in wearable devices and artificial intelligence present unprecedented opportunities to transform neurological care delivery. Consumer-grade devices now enable the continuous collection of objective physiological measurements [10,11], while large language models demonstrate remarkable capabilities in medical data interpretation and communication [12]. However, the integration of these technologies into clinically validated, accessible platforms specifically designed for brain-related disease management remains largely unexplored, representing a significant opportunity for innovation in digital health.

This paper introduces PARKA AI, a novel mobile health platform that addresses these gaps through the systematic integration of sensor-derived measurements, patient-reported outcomes, and LLMs. Our approach proposes a unified system that supports both patient independence and clinical decision making through advanced human–computer interaction principles [13,14].

The research presented herein makes several important contributions to the field of digital health and neurological care:

- We design and implement an integrated patient–provider communication framework specifically for PD management.
- We develop accessibility-centered interfaces that accommodate motor and cognitive difficulties characteristic of PD.
- We implement an LLM-powered clinical reporting system that enhances workflow efficiency while maintaining medical accuracy.
- We demonstrate measurable improvements in patient engagement and clinical decision making through comprehensive use case walk-throughs.

Together, these elements position PARKA AI as a single system that spans sensing, self-reports, and LLM-mediated, role-aware communication. Our approach is the first step towards a scalable technological framework for continuous neurological care coordination that bridges self-management and professional oversight. Through systematic development and implementation, this work demonstrates how emerging technologies can be thoughtfully integrated to address real-world challenges in chronic disease management while maintaining focus on patient needs and practicality. Distinct from prior PD apps, PARKA AI uniquely integrates (i) continuous Apple HealthKit sensor streams, (ii) structured self-reports, and (iii) LLM-generated outputs that bifurcate into lay summaries for patients and templated, citation-friendly reports for clinicians, all within accessibility-centered interfaces.

The remainder of this paper is organized as follows: Section 2 surveys related work across four areas—digital biomarkers, generative AI in healthcare, patient–provider communication, and health data visualization. Section 3 details our approach, outlining design requirements and the prototype implementation. Section 4 presents demonstrative use cases, Section 5 discusses implications and future work, and Section 6 concludes.

## 2. Related Work

We frame related work across four areas: (i) digital biomarkers from wearables/phones for PD detection, (ii) generative AI usage in healthcare, (iii) technologies for patient–provider communication, and (iv) health data visualization. Together, they show progress in sensing and explanation, yet few systems fuse sensor and self-reported data with AI-generated, dual-audience summaries embedded in clinical workflow. The subsections review each area and distill requirements that shaped PARKA AI.

### 2.1. Digital Biomarkers for Parkinson’s Disease

Wearable technologies have significantly advanced the monitoring of Parkinson’s disease (PD) by enabling the continuous, objective assessment of motor and non-motor symptoms through digital biomarkers. Devices like the Apple Watch, equipped with accelerometers and gyroscopes, capture metrics such as tremor frequency, gait variability, and sleep patterns with high precision, as demonstrated by large-scale studies like the IDEA-FAST consortium [15]. For instance, research has shown that wearable-derived metrics, such as step count and tremor amplitude, correlate strongly with clinical assessments, enabling data-driven treatment adjustments [16]. The WATCH-PD study, a multi-center initiative, further validated the use of Apple Watch sensors for detecting early PD progression, achieving sensitivity rates above 85% for tremor detection [10]. Despite these advancements, most wearable-based tools remain research-focused or clinician-oriented, lacking user-friendly interfaces that empower patients to self-manage their condition or share real-time data with healthcare providers (HCPs) [10]. This gap in patient-centric design limits the practical utility of digital biomarkers in daily PD management, a challenge PARKA AI seeks to address.

### 2.2. Generative AI in Healthcare

Generative artificial intelligence (AI) models have emerged as powerful tools for simplifying complex medical information, enhancing patient understanding and engagement. Models like Google Gemini 1.5 Flash have demonstrated the ability to generate clear, concise summaries of medical data, with applications in patient education and clinical decision support [12]. In the context of PD, tools like Patrika employ conversational AI to facilitate symptom tracking and provide educational content, achieving user satisfaction rates of up to 78% in preliminary studies [17]. However, these tools often rely on patient-reported inputs without integrating real-time sensor data, limiting their ability to provide comprehensive insights [17]. Google Gemini 1.5 Flash, validated in healthcare settings for its ability to produce lay-language summaries with 92% readability scores on standardized metrics, offers a robust solution for translating PD-related sensor data into actionable insights for both patients and HCPs [12]. PARKA AI leverages this capability to bridge the gap between sensor-driven data and user-friendly communication.

### 2.3. Patient-Provider Communication Technologies

Effective communication between patients and HCPs is critical for improving treatment adherence and outcomes in PD, yet it is often hindered by time constraints, complex medical terminology, and cognitive impairments [5]. Digital tools like Talk2Care utilize AI to enable asynchronous communication, reducing the burden of in-person visits and achieving a 30% improvement in patient-reported satisfaction in chronic disease management [18]. However, such tools are typically designed for general telehealth applications and lack specific adaptations for PD’s unique needs, such as integrating real-time motor and non-motor symptom data [18]. Studies emphasize that PD patients benefit from tools that provide structured, data-driven insights to facilitate discussions with HCPs, yet no existing platform fully integrates wearable data with AI-driven communication [5,10]. PARKA AI addresses this by combining real-time HealthKit data with AI-generated summaries, enabling seamless patient–HCP interaction tailored to PD.

### 2.4. Data Visualizations for Health

Data visualizations play a pivotal role in making complex health data accessible to both patients and clinicians. Systems like Lifelines, which visualizes longitudinal EMR data, have reduced clinicians’ data interpretation time by up to 25% in controlled studies [19]. However, these tools are designed for HCPs and lack patient-oriented interfaces, rendering them inaccessible to individuals with cognitive or motor impairments [20]. In PD, tools like ArmSleeve focus on visualizing upper limb motor symptoms using wearable sensors but neglect non-motor symptoms like mood or sleep, which are critical for holistic management [21]. Recent stroke rehabilitation visualization research highlights the need for patient-friendly, skeuomorphic designs that accommodate cognitive challenges, achieving up to 40% higher user engagement in usability studies [22]. PARKA AI builds on these principles, offering comprehensive visualizations that integrate motor and non-motor data, tailored for both patients and HCPs.

### 2.5. Comparison with Existing PD Tools

Research-focused platforms such as the WATCH-PD study [23] demonstrate that Apple Watch and smartphone sensors can longitudinally assess early PD motor function. However, outputs are primarily clinician-facing and not optimized for day-to-day patient self-management. In parallel, commercial PD monitoring devices emphasize sensor-derived symptom tracking but often surface findings through dashboards oriented to healthcare professionals, offering limited support for patients’ daily use and cognitive accessibility needs [9]. By contrast, conversational journaling approaches such as Patrika leverage LLMs to support education and symptom logging, yet they do not integrate continuous wearable data streams and, thus, miss objective context for day-to-day trends [17]. Screening-oriented work that applies LLMs to self-reported questionnaires is promising for detection, but it is episodic and not designed for ongoing management or clinical reporting workflows in PD [8].

Other tools, such as Talk2Care [18], emphasize asynchronous communication between patients and providers, improving access to care but offering only generic messaging functions without tailoring to Parkinson’s disease or integrating sensor and self-reported data. By contrast, PARKA AI proposes to bring these strands together in a unified system: Tt integrates continuous HealthKit data with daily self-reports, applies an LLM to generate role-specific outputs for both patients and clinicians, and incorporates accessibility features tailored to the motor and cognitive challenges of PD. This combined design aims to provide an interface for patients to receive comprehensible, supportive insights for self-management, while clinicians may benefit from structured summaries embedded in familiar workflows. In this way, our proposed approach, PARKA AI, aims beyond existing tools by spanning sensing, journaling, education, and clinical reporting within a single, patient-centered platform.

## 3. Our Approach: PARKA AI

### 3.1. Design Requirements

Through a human-centered design approach [13], informed by a literature review [10,15,16] and stroke rehabilitation visualization principles [22], we derived five design requirements for PARKA AI:

- Presenting a holistic view of patient progress that consolidates motor (tremors and mobility) and non-motor (mood and sleep) data to streamline EMR navigation;
- Categorizing assessments by health domains to organize sensor-derived biomarkers and self-reported data for intuitive access;
- Providing periodic progress reports, particularly weekly summaries to foster meaningful patient–HCP discussions;
- Using simple, lay language to ensure comprehension for patients with cognitive impairments;
- Using accessible visualizations, leveraging intuitive charts and skeuomorphic elements to enhance usability across diverse users.

### 3.2. Prototype Implementation

**Figure 1:**
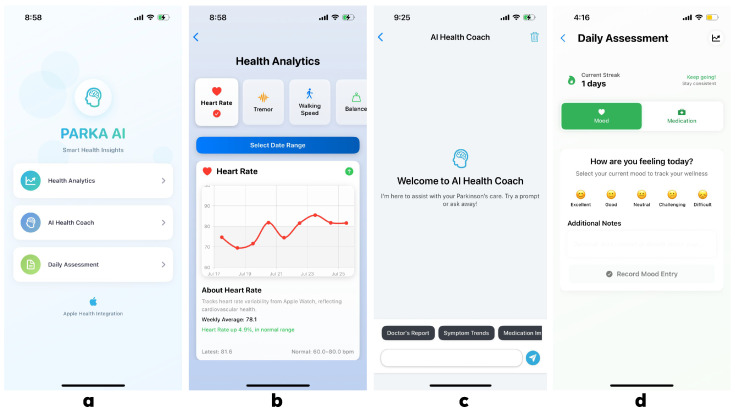
PARKA AI application interface components: (**a**) homepage interface providing centralized navigation and dashboard overview with integrated HealthKit data visualization, (**b**) HealthAnalytics module featuring MetricChartView with scrollable parameter selector, haptic feedback navigation, and zoomable line charts for symptom tracking and clinical metrics visualization, (**c**) AICoach interface powered by Google Gemini 1.5 Flash delivering personalized educational content and coaching recommendations through an accessible chat interface and PDFPreviewView for generating editable healthcare provider reports, and (**d**) DailyAssessment component incorporating SelfReportView for mood and medication logging with motivational reminders.

This manuscript presents PARKA AI, a high-fidelity prototype. No clinical deployment with real patients was conducted here, and validation is planned as future work. PARKA AI was developed as an iOS application using Swift and Xcode, integrating the following data that can be collected through Apple Watch and is accessible through Apple HealthKit, which functions as a central repository for health and fitness data: tremor intensity, step count, walking speed, heart rate, and sleep duration. These data are paired with self-reported logs through daily questionnaires for mood and medication logging. The application features a centralized homepage interface that provides navigation access to all modules (Figure 1a). Google Gemini 1.5 Flash generates lay-language summaries and structured HCP reports, leveraging its high readability score for PD-related content [12]. The app adheres to the Visual Information Seeking Mantra [14], offering an overview, zoom and filter options, and details on demand.

Key components include MetricChartView within the HealthAnalytics module, displaying line charts with a scrollable parameter selector and haptic feedback for motor-impaired users (Figure 1b); AICoach module, delivering educational content via a Gemini-powered chat interface (Figure 1c); and DailyAssessment module for medication and mood logging (Figure 1d), where collected data is processed by our LLM-powered AI Coach to generate comprehensive doctor reports through PDFPreviewView. Accessibility features include 16 pt font, zoomable charts, and colorblind-safe blue/white schemes. The prototype was rigorously tested on an iPhone emulator and a physical device.

## 4. Demonstrative Use Cases

PARKA AI’s potential is vividly showcased through three compelling use cases, each highlighting its ability to empower diverse PD patients and HCPs in real-world scenarios. These cases demonstrate the intuitive design, accessibility, and impact of the application on self-management and clinical decision making, underscoring its value in addressing the multifaceted challenges of PD.

### 4.1. Use Case 1: Empowering Self-Management Through Symptom Tracking

**Figure 2:**
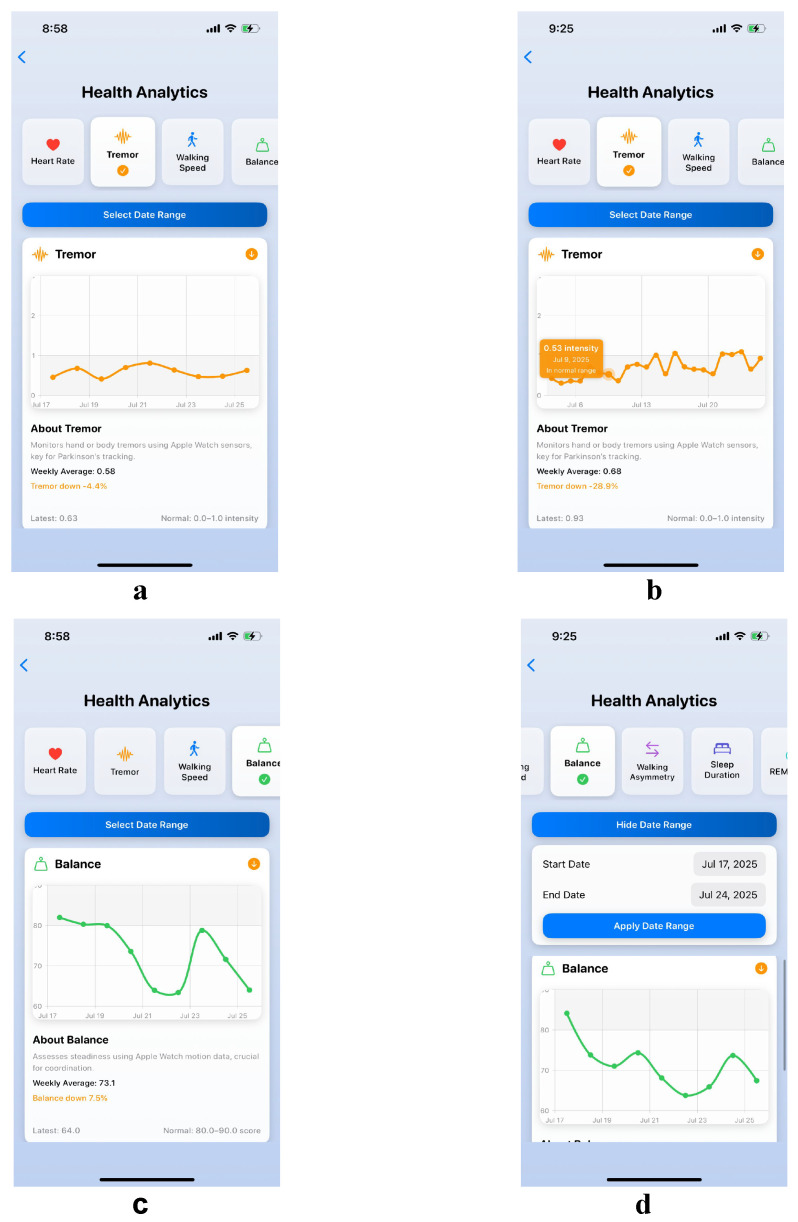
PARKA AI dual interface architecture for Parkinson’s disease management. (**a**) MetricChartView showing tremor intensity monitoring with haptic feedback navigation for motor-impaired users. (**b**) Data point detail view displaying tremor measurements within normal range validated by HealthKit integration. (**c**) Balance monitoring interface revealing concerning gait variability increase over two weeks with accessible design features including zoomable charts and 16-point fonts. (**d**) MetricDetailsView used by clinicians for temporal correlation analysis, featuring date range picker to identify medication-related symptom onset patterns.

This use case demonstrates PARKA AI’s dual interface architecture through the interconnected care journey of Lily, a 55-year-old music teacher with early-stage Parkinson’s disease, and her neurologist Dr. N. Lily relies on MetricChartView to monitor her tremor intensity, navigating the scrollable parameter selector with haptic feedback that confirms selections despite motor impairments (Figure 2a). The line chart displays stable tremor trends between 2.0 and 2.5 on a 0 to 5 scale, and tapping data points reveals her symptoms are within normal range as validated by HealthKit data (Figure 2b), reassuring her that therapy is effective and boosting confidence for professional activities.

However, balance monitoring captures a concerning increase in gait variability over two weeks (Figure 2c), which Lily identifies through the accessible interface design featuring zoomable charts and 16-point fonts. During their 15 min consultation, Dr. N uses MetricDetailsView to assess this balance decline, employing the date range picker to correlate the symptom onset with a medication adjustment made three weeks prior (Figure 2d). All of these monitoring data, including tremor stability measurements, balance variability patterns, and timeline correlations, are automatically fed into the LLM system, enabling comprehensive clinical report generation that synthesizes objective sensor data with temporal analysis for evidence-based treatment planning.

This data-driven correlation enables evidence-based treatment modifications including dopamine agonist dosage adjustment and balance-focused physical therapy, reducing decision-making time compared to traditional EMR reviews. The integration between patient self-management and clinical oversight creates a continuous care loop that identifies medication-related issues proactively, preventing potential functional decline while maintaining treatment precision through familiar clinical workflows and automated trend analysis enhanced by AI-powered data synthesis for comprehensive clinical documentation.

### 4.2. Use Case 2: Enhancing Patient Education via AI-Driven Insights

**Figure 3:**
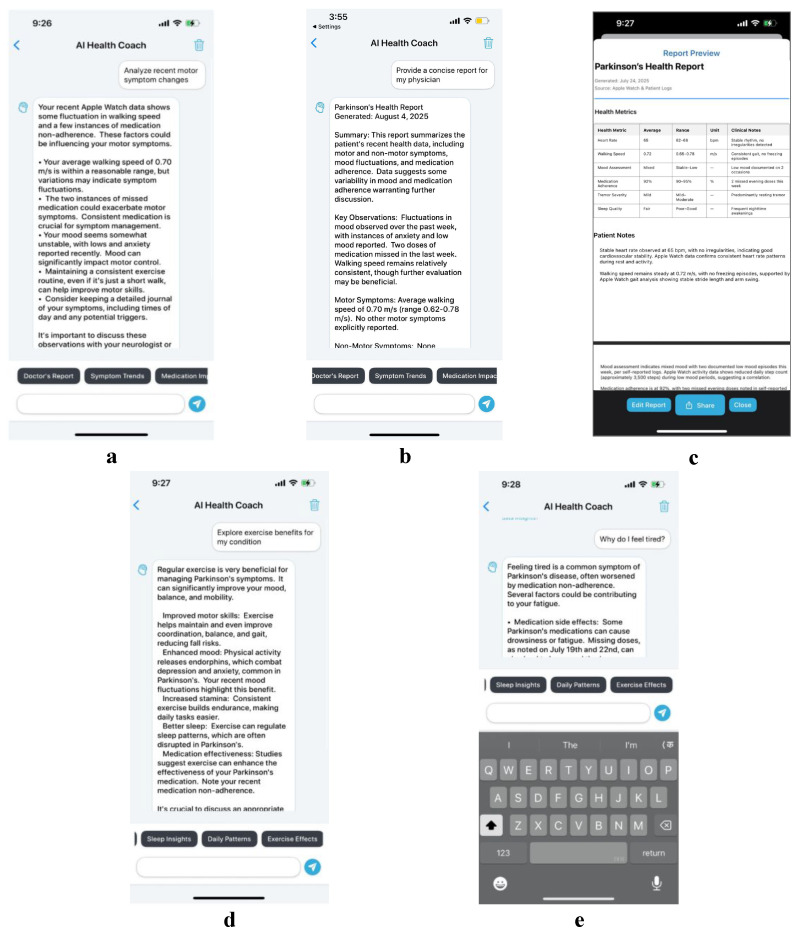
AI Coach Interface: (**a**) concise motor-symptom analysis generated from recent HealthKit trends (“Symptom Trends”), (**b**) clinician-oriented summary produced via the “Doctor’s Report” prompt, (**c**) professional report preview suitable for medical documentation, (**d**) patient-education response explaining exercise benefits, and (**e**) patient free-text query (“Why do I feel tired?”) shown with simplified AI explanation. The revised figure reduces textual load and emphasizes clear, visual interaction flow.

This use case demonstrates PARKA AI’s educational capabilities through the interconnected experiences of Maria, a newly diagnosed Parkinson’s patient, and Dr. P, her treating neurologist, showcasing how AI-driven insights bridge the gap between patient understanding and clinical expertise. Maria, a 60-year-old retiree newly diagnosed with Parkinson’s disease, begins her journey with AICoachView to understand the fatigue that has been affecting her daily activities. Upon accessing the interface, she encounters an engaging brain icon and scrolling quick prompt bar that makes complex medical information approachable, as shown in the initial chat interface (Figure 3a).

When Maria clicks on “Symptom Trends” from the prompt options, the system generates a contextual prompt stating “Analyze recent motor symptom changes”, which appears in the chat interface (Figure 3a). Each time she selects this option, the AI provides related but varied prompts to help her observe different aspects of her symptom patterns, ensuring comprehensive understanding through diverse analytical perspectives. The system presents motor change analysis with precautionary notifications displayed prominently below responses, ensuring she understands both the insights and safety considerations (Figure 3a). Motivated to learn more, Maria types personal questions such as “Why do I feel tired?” and the chat interface displays her query with the AI preparing a personalized response (Figure 3e). She also requests exercise recommendations, and the AI coach responds with accessible explanations about sleep disruptions and tailored activity suggestions as demonstrated in the conversational flow (Figure 3d). This educational foundation empowers Maria to engage more meaningfully with her healthcare team and take active steps in her care management.

During Maria’s follow-up appointment, Dr. P leverages the same AICoachView system to generate comprehensive clinical insights that build upon Maria’s self directed learning. When Dr. P selects the “Doctor’s Report” prompt from the clinical interface, the system begins generating professional medical content (Figure 3b). Google Gemini 1.5 Flash accesses Maria’s continuous monitoring data, the same information that informed her educational interactions, and produces a structured medical summary including heart rate patterns, walking speed trends, and medication adherence rates, as shown in the clinical report generation process (Figure 3b,c). The completed clinical summary appears in a professional format suitable for medical documentation (Figure 3c).

The clinical chat interface presents this information using professional medical terminology while drawing from the identical data sources that powered Maria’s patient-education experience. The AI-generated report transitions to PDFPreviewView (Figure 3c), where Dr. P adds personalized therapy recommendations that directly address the concerns Maria raised in her self-directed learning sessions. The integration creates a powerful feedback loop where Maria’s AI-assisted learning directly informs clinical decision making. When Maria returns home, she can continue exploring topics that Dr. P discussed during their appointment, with the AI coach providing consistent, evidence-based information that reinforces clinical recommendations.

Dr. P benefits from patients who arrive more informed and engaged, reducing consultation time spent on basic education while enabling deeper discussions about treatment optimization. This relationship demonstrates how AI-driven insights can simultaneously empower patient self-management and enhance clinical efficiency, with both users benefiting from the same underlying data intelligence adapted to their respective needs and expertise levels. This reduction in clinical report generation time allows Dr. P to spend more quality interaction time with informed patients like Maria, ultimately improving care outcomes through enhanced patient–clinician collaboration.

### 4.3. Use Case 3: Supporting Adherence Through Mood and Medication Tracking

**Figure 4:**
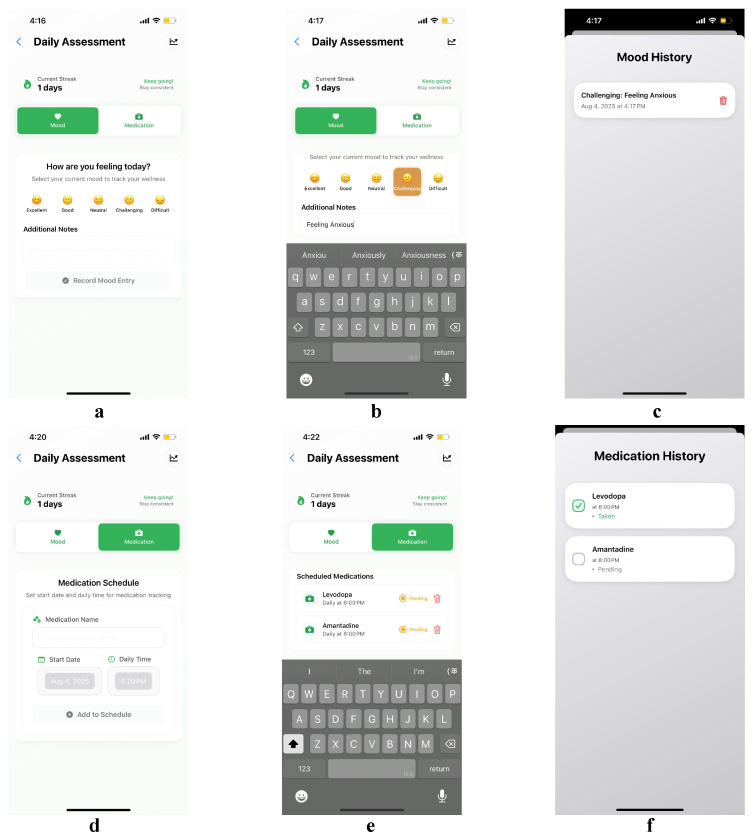
DailyAssessment interface: (**a**) mood-selection interface with various emotional states, (**b**) detailed mood logging with anxiety notes and contextual information, (**c**) comprehensive mood history with patterns and trends over time, (**d**) motivational streak notifications with positive reinforcement messages, and (**e**) medication-logging interface with Levodopa and Amantadine dose recording and haptic feedback, (**f**) automatically generated daily tracking entries displayed as a checkbox tasks for taking medications.

This use case illustrates PARKA AI’s self management capabilities through Elena’s daily routine of mood and medication tracking, demonstrating how streamlined scheduling and motivational features enhance treatment adherence and emotional awareness. Elena, a 58-year-old librarian with Parkinson’s disease, uses the DailyAssessment feature to monitor her psychological and medical status. In the mood-tracking interface, Elena selects from various emotional states (Figure 4a). On a challenging day, she chooses “Challenging” and adds notes about her anxiety, providing context for future therapy discussions (Figure 4b). The system stores a comprehensive mood history, enabling Elena to review patterns and identify triggers related to her condition (Figure 4c). To promote consistent tracking, PARKA AI uses streak notifications and positive reinforcement messages like “Keep it up!” to track consecutive logging days. Gentle end-of-day reminders encourage voluntary logging without feeling intrusive, leveraging the streak feature to motivate Elena.

The medication-tracking component integrates seamlessly with mood monitoring to support comprehensive daily management. Elena sets up her Levodopa (a dopmanine replacement drug for PD) regimen once, specifying a daily 6 PM dose, and the system automatically generates a daily tracking entry displayed as a checkbox task (Figure 4f). The medication history shows only today’s scheduled dose with a clear time indicator. At the end of each day, Elena marks the dose as taken or missed using a simplified three-state checkbox system (pending/taken/missed) with large touch targets and color-coded indicators to accommodate her motor difficulties and provide immediate visual feedback. Each morning, a new entry for the current day’s dose appears, and the previous day’s entry is archived. The archived medication data, along with the previous day’s mood data, are automatically sent to the LLM for integration into a clinical report, ensuring doctors receive a comprehensive summary of Elena’s medication adherence and emotional patterns.

Over a week, Elena achieves 100% medication adherence, as tracked by the system’s rolling daily entry approach, which presents a clean slate each day while maintaining comprehensive logs in the background. This streamlined workflow requires only simple checkbox interactions, reducing cognitive load. When Elena generates a clinical report via the AI coach interface, the LLM combines her mood history and medication adherence data, including daily checkbox statuses and mood entries, to produce detailed summaries of her emotional and treatment consistency. This integrated reporting provides doctors with a complete view of Elena’s psychological and medical status, enabling informed treatment decisions that address both physical and mental health.

The combination of mood and medication tracking creates a useful self-management tool, empowering Elena to maintain emotional clarity and treatment consistency through a simplified workflow. The motivational streak system transforms routine compliance into an engaging practice, while the rolling daily entry system minimizes interface complexity by focusing on immediate medication needs. This approach highlights PARKA AI’s potential effectiveness in supporting Parkinson’s disease management through user-centered design, prioritizing medical necessity, cognitive accessibility, and patient autonomy via intelligent automation and simplified interactions.

## 5. Discussion and Future Work

PARKA AI can potentially enhance how people manage Parkinson’s disease by bringing together iPhone HealthKit data, personal symptom logs, and AI powered insights in one easy-to-use platform. Our design offers two different views: simple, familiar interfaces that patients find comfortable to use, and detailed clinical formats that healthcare professionals need [22,24]. The app tackles a major challenge for PD patients making sense of complex medical information by offering personalized filters and explanations in everyday language that anyone can understand [25]. This approach builds on health literacy research to make disease management less stressful and more empowering.

Growing external evidence supports the use of consumer wearables and smartphones to derive reliable digital biomarkers for PD. Recent multi-center work shows that sensor-derived measures of gait, tremor, tapping, and speech can differentiate early, untreated PD from controls and track progression, while reviews synthesize strong potential for precise monitoring of motor and sleep-related symptoms [10]; smartwatch-based tremor detection studies report discriminative performance (e.g., AUCs approximately 0.7–0.8) [23]. These findings reinforce PARKA AI’s emphasis on integrating HealthKit data streams, which include sensor-derived biomarkers and highlight the need for device calibration, test–retest reliability, and cross-device harmonization in future deployments

Our current testing uses simulated patient data, which gives us a strong starting point for real-world studies with actual patients and doctors. As the next critical step, we plan to move beyond simulation and conduct pilot usability studies with Parkinson’s patients and their healthcare providers. These studies will evaluate the app’s reliability, patient adherence, validity, and clinical utility using standardized assessment tools, ensuring that future iterations meet the highest standards of objectivity and reproducibility across neurological pathologies. Future work includes working with healthcare providers to test usability and conducting long-term studies with patients to see how well our iPhone integration and AI analysis perform in practice. To promote adoption and enable collection of diverse, real-world datasets, we will also disseminate PARKA AI through standard app distribution platforms (e.g., Apple TestFlight and App Store). This will allow both patients and healthcare professionals to access the tool directly, accelerating feedback, dataset generation, and broader validation. We plan to develop smarter AI that adapts to each patient’s unique needs, make the app available across different devices using advanced deployment technology [26], and scale our solution to help Parkinson’s patients.

To translate usability into clinical impact, subsequent studies should align with emerging regulatory expectations for digital health technologies (DHTs) used in clinical investigations, including validity, data integrity, and risk management [27]. This would require robust privacy protections which were not included in this work, as we utilized an open-source LLM (Google Gemini 1.5 Flash). One workaround could be the use of on-device, compact LLMs (e.g., TinyLlama [28]) that can be further trained on medical use cases and that do not transmit any data to third parties. Future work including production deployment will follow HealthKit data-use restrictions and applicable health-data laws (e.g., HIPAA/PHIPA). We will implement least-privilege access, on-device preprocessing when feasible, encryption in transit/at rest, and auditable consent flows. Clinician-in-the-loop controls will require human review before AI-generated text is added to records; summaries will include inline provenance (metrics/date ranges) and safety disclaimers. We will log prompts/outputs for auditability, use templates for critical sections (medications and red-flags), conduct pre-release bias/error testing, and maintain an incident-response process. Regulatory alignment will follow FDA/Health Canada DHT guidance emphasizing validity, data integrity, and risk management.

Evidence from randomized and national trials of telemedicine in PD demonstrates feasibility and comparable outcomes versus in-person care, suggesting that PARKA AI’s auto-synthesized summaries could further streamline virtual visits [29]. Parallel work shows LLMs can reliably simplify clinical text into patient-friendly language, improving readability, supporting our patient-facing reporting goals [30]. PARKA AI represents an important step in integrating generative AI with objective sensor-derived metrics for self-management and HCP–patient communication for PD.

## 6. Conclusions

PARKA AI presents an innovative approach to Parkinson’s disease management, seamlessly integrating Apple Watch HealthKit data, self-reported logs, and Google Gemini 1.5 Flash insights into a high-fidelity iOS application. Its user-friendly visualizations, personalized tools, and actionable HCP reports empower patients to proactively keep track of their ailment and streamline clinical workflows. The compelling use cases vividly demonstrate its potential to bridge communication gaps, making it a useful and portable tool for PD care. Future work includes building robust privacy protections in PARKA AI and real-world validation through longitudinal studies in the wild, to assess its role in treatment outcomes and quality of life for PD patients.

## Data Availability

The data presented in this study are available on request from the corresponding author.
